# A Novel Method to Predict Mortality and Length of Stay after Transfemoral Transcatheter Aortic Valve Implantation

**DOI:** 10.3390/medicina57121332

**Published:** 2021-12-06

**Authors:** Maria Zisiopoulou, Alexander Berkowitsch, Philipp Seppelt, Andreas M. Zeiher, Mariuca Vasa-Nicotera

**Affiliations:** Department of Cardiology, University Hospital Frankfurt, Theodor-Stern-Kai 7, 60590 Frankfurt am Main, Germany; Alexander.Berkowitsch@kgu.de (A.B.); philipp.seppelt@kgu.de (P.S.); zeiher@em.uni-frankfurt.de (A.M.Z.); mariuca.vasa-nicotera@kgu.de (M.V.-N.)

**Keywords:** aortic valve stenosis, TAVI, outcomes, KCCQ, EQ5D5L, biomarkers, prediction, personalized

## Abstract

*Background and Objectives*: We tested if a novel combination of predictors could improve the accuracy of outcome prediction after transfemoral transcatheter aortic valve implantation (TAVI). *Materials and Methods*: This prospective study recruited 169 participants (49% female; median age 81 years). The primary endpoint was midterm mortality; secondary endpoints were acute Valve Academic Research Consortium (VARC)-3 complication rate and post-TAVI in-hospital length of stay (LoS). EuroSCORE II (ESII), comorbidities (e.g., coronary artery disease), eGFR (estimated glomerular filtration rate; based on cystatin C), hemoglobin, creatinine, N-Terminal pro-Brain Natriuretic Peptide (NTproBNP) levels and patient-reported outcome measures (PROMs, namely EuroQol-5-Dimension-5-Levels, EQ5D5L; Kansas City Cardiomyopathy Questionnaire, KCCQ; clinical frailty scale, CFS) at baseline were tested as predictors. Regression (uni- and multi-variate Cox; linear; binary logistic) and receiver operating characteristic (ROC)-curve analysis were applied. *Results*: Within a median follow-up of 439 (318–585) days, 12 participants died (7.1%). Independent predictors of mortality using multivariate Cox regression were baseline eGFR (*p* = 0.001) and KCCQ (*p* = 0.037). Based on these predictors, a Linear Prediction Score (LPS1) was calculated. The LPS1-area under the curve (AUC)-value (0.761) was significantly higher than the ESII-AUC value (0.597; *p* = 0.035). Independent predictors for LoS > 6 days (the median LoS) were eGFR (*p* = 0.028), NTproBNP (*p* = 0.034), and EQ5D5L values (*p* = 0.002); a respective calculated LPS2 provided an AUC value of 0.677 (*p* < 0.001). Eighty participants (47.3%) experienced complications. Male sex predicted complications only in the univariate analysis. *Conclusions*: The combination of KCCQ and eGFR can better predict midterm mortality than ES II alone. Combining eGFR, NTproBNP, and EQ5D5L can reliably predict LoS after TAVI. This novel method improves personalized TAVI risk stratification and hence may help reduce post-TAVI risk.

## 1. Introduction

Trans-catheter aortic valve implant (TAVI) procedures have become the mainstream therapy of degenerative aortic valve stenosis not only in elderly ineligible or poor surgical candidates but increasingly in patients with moderate surgical risk. Although the TAVI-related one-year mortality in Western Europe has dropped considerably, a one-year mortality rate as high as 19.9% was reported in 2015 [1]. In-hospital mortality after TAVI has been significantly reduced in 2014 (4.2%) compared with 2008 (10.4%) [2], but 30-day mortality still is 7%, and one-year all-cause mortality may range between 11% [3] and 22.7% for patients with low-flow low-gradient aortic stenosis and even reach 24.1% for patients with moderate/severe impaired left ventricular function [4]. Moreover, the rate of acute (up to 30 days) TAVI complications is 23.1% [5].

As a result, it is of paramount importance to identify patients at increased risk of complications and/or mortality before the TAVI procedure (at baseline) in order to (a) provide personalized pre-interventional risk stratification and hence optimize patient consultation and involvement and (b) adapt and focus the clinical management pathways accordingly to meet the expected increased post-interventional risk and, therefore, increase safety in high-risk patients in a targeted approach. Currently, the performance to predict outcome using existing TAVI-CPMs (clinical prediction models), including EuroSCORE II (ESII), is rather low [6].

Moreover, the patients’ perspective may provide additional information regarding final clinical decisions based on the patients’ preference [7]. Patient-reported outcome performance measures have been shown to capture the aspects of healthcare that are most important and relevant for the patients [8]. Nonetheless, there are few studies that use patient-reported outcome measures (PROMs) to provide risk stratification not only from the clinicians’ but also from the patients’ point of view. In this study, we tested if PROMs could provide important additional information for risk stratification both from the patient and the clinical point of view for patients with significant aortic stenosis that are considered as TAVI candidates.

Additionally, attention is also increasingly focusing on improvement of TAVI-related in-hospital pathways. One important parameter describing the quality of the TAVI clinical process is post-procedural in-hospital length of stay (LoS). For instance, the average LoS for TAVI patients in Germany in 2020 was 11.5 days, while studies in other countries report significantly lower LoS [9,10]. Prolonged hospital stay is associated with an increase in hospital-acquired infections and other complications [11]. To maintain the high standard of quality of patient care despite increasing numbers of TAVI interventions at specialized medical centers, a more standardized approach to clinical quality improvement programs is required. PROMs could provide significant information to achieve this additional goal.

Regarding the predictive value of biomarkers, high levels of baseline N-Terminal pro-Brain Natriuretic Peptide (NTproBNP) have been associated with a significant increase in midterm (6-month to 4-year) mortality (pooled odds ratio/hazard ratio, OR/HR, 1.88) and a non-significant increase in early (30-day or 2-month) mortality (pooled OR, 1.60) [12]. In addition, in stenotic aortic valves, cystatin C mRNA (messenger ribonucleic acid) and protein levels are increased [13]. Hence, it is reasonable to expect that increased baseline levels of cystatin C and reduced estimated glomerular filtration rate (eGFR) values based on cystatin C levels may predict an adverse outcome (including mortality) after TAVI. Given that anemia has been associated with overall mortality (HR 2.40) [14], the baseline blood hemoglobin level may be an additional relevant candidate biomarker for TAVI outcome.

To date, Kansas City Cardiomyopathy Questionnaire (KCCQ), a questionnaire developed specifically to monitor heart failure outcomes, has been used in only a few studies to assess TAVI outcomes [15,16,17]. Moreover, there are no reports on studies that were prospectively designed to test KCCQ in combination with other patient-reported outcomes and/or clinical scoring systems or biomarkers as predictors of TAVI outcomes. Additionally, no study assessed the value of prospectively studied patient-reported EuroQol-5 Dimension-5 Levels (EQ5D5L) questionnaire values as a predictor of TAVI outcomes.

For these reasons, in this study, we investigated if a combination of baseline patient-reported outcome measures (PROMs) such as KCCQ and EQ5D5L and clinical scoring systems, comorbidities, and the aforementioned biomarkers could improve the prediction of TAVI outcomes. Our target was to promote patient-centered care and safety and provide a knowledge foundation for informed decisions from both the patients’ as well from the clinicians’ perspective regarding expectations and performance of TAVI procedures.

## 2. Materials and Methods

All patients who underwent a trans-femoral TAVI procedure from January 2019 until March 2020 in the cardiology department of our tertiary university hospital were asked to participate in this pilot prospective study.

### 2.1. Inclusion Criteria

The main inclusion criteria were a severe symptomatic degenerative aortic stenosis with an effective orifice area (EOA) < 1.0 cm^2^ or mean gradient > 40 mmHg and a NYHA (New York Heart Association) functional class equal or greater than II. The final decision to implant was made by the local Heart Team Board on an individual personalized basis.

### 2.2. Exclusion Criteria

Patients presenting with acute cardiogenic shock or hemodynamic instability requiring inotropic support prior to TAVI, and patients with severe neurological disorders, dementia, or inability to provide informed consent due to their mental condition were excluded. Furthermore, patients with an existing aortic prosthetic heart valve requiring a valve-in-valve procedure were not included.

All investigative procedures (i.e., PROMs, clinical scoring systems, blood testing) were performed in accordance with the Declaration of Helsinki for studies in humans at admission to the hospital one day prior to intervention. Written informed consent was given by all participants. The study was approved by the local Institutional Review Board (Ethics Committee of the Faculty of Medicine; Nr. 296/16).

In order to standardize TAVI-related data acquisition, we have conceived and implemented a TAVI Scorecard. On this scorecard, we collected at baseline EuroSCORE II (ESII), comorbidities (see Table 1), blood hemoglobin, creatinine, and NTProBNP levels, eGFR (based on cystatin C) as well as EuroQol-5 Dimension-5 Levels (EQ5D5L), Kansas City Cardiomyopathy Questionnaire (KCCQ), and clinical frailty scale (CFS).

### 2.3. Collected Data

#### 2.3.1. EuroSCORE II

EuroSCORE II is an established clinical scoring system to predict operative mortality from cardiac surgery. EuroSCORE II was determined prior to intervention for each patient with a web-based calculator (http://euroscore.org/calc.html; accessed between 1 January 2019 and 31 March 2020). [18] However, ES II does not include potentially relevant risk factors such as certain comorbidities (e.g., atrial fibrillation) or biomarkers such as blood levels of NTproBNP and eGFR (based on cystatin C).

#### 2.3.2. Comorbidities

Clinically significant comorbidities are presented on Table 1.

#### 2.3.3. Laboratory Investigations/Biomarkers

Baseline eGFR (based on cystatin C), blood hemoglobin, creatinine, and NTproBNP levels have been measured in samples obtained from the participants one day before the TAVI procedure.

#### 2.3.4. EuroQol-5 Dimension-5 Levels (EQ5D5L)

The international questionnaire EuroQol-5 Dimension-5 Levels (EQ5D5L) is a standardized measurement of the quality of life (QoL) of a patient [19]. It consists of questions that assess QoL on the current day. The first part involves five questions on mobility, self-care, usual activities, pain/discomfort, and anxiety/depression, which are defined in five dimensions (no/slight/moderate/severe/extreme problems) for any question. The second part is the EQ Visual Analog Scale, which is a self-rated health scale and ranges from 0 to 100; 100 means the best and 0 means the worst health that the patient can imagine [20].

#### 2.3.5. Kansas City Cardiomyopathy Questionnaire (KCCQ)

The Kansas City Cardiomyopathy Questionnaire (KCCQ) is a sensitive, specific, and responsive health-related QoL measure for patients with heart disease [21,22]. The KCCQ is a 23-item, self-measurement instrument with four domains: (1) “Physical limitation” (PhyLi), (2) “Symptom frequency” (SymFre), (3) “Quality of life” (QoL), and (4) “Social limitation” (SoLi).

#### 2.3.6. Clinical Frailty Scale (CFS)

The CFS is a simple scale with a score ranging from 1 (very fit, performing sport activities on a regular basis) to 9 (terminally ill, with an estimated life expectancy of less than 6 months). The answers are based on descriptors and pictographs of activity and functional status. The patient is asked to tick in one of the nine boxes that describe their level of frailty in everyday domestic life [23,24].

### 2.4. Intervention—TAVI Procedure

All participants were treated under conscious sedation. The TAVI procedure was performed as per standardized operating procedure protocols for balloon-expandable and self-expandable prostheses.

### 2.5. Clinical Endpoints

#### 2.5.1. Primary Endpoint: Midterm Mortality

All-cause midterm mortality was used as the primary endpoint. The time frame included mortality that occurred between 3 months and 2 years after the TAVI procedure.

#### 2.5.2. Secondary Endpoints

Secondary endpoints were (1) acute VARC-3 (Valve Academic Research Consortium-3) complication rate and (2) in-hospital length of stay (LoS). The clinical endpoints stroke, bleeding complications, acute kidney injury, vascular complications, conduction disturbances, and arrhythmias were assessed as defined by the standardized and updated VARC-3 criteria [25].

### 2.6. Statistical Analysis

This study was designed as a pilot study. Primary endpoint (i.e., midterm mortality after trans-femoral TAVI) analysis was performed using univariate and multivariate Cox regression models. The secondary endpoints were analyzed with univariate and multivariate binary logistic regression. In multivariate analyses, only variables that were significantly associated with each endpoint in the univariate analysis were included. Additionally, a linear predictor score (LPS; LPS1 for primary endpoint, LPS2 for secondary endpoints) was calculated by performing multivariate analysis. Calculation of LPS is described by Formula (1)

LPS = ∑BiXi(1)

where Xi is the independent predictor and Bi is the regression coefficient of this predictor as calculated in multivariate regression analysis. Receiver operating characteristic (ROC) curve analysis was also performed, where appropriate. Society for Thoracic Surgery (STS) scores were not included in the analysis, due to restrictions placed by the multivariate analysis and the non-availability of its formula. The data were presented as number (percentage) or median (interquartile range, IQR). The differences were considered significant by error probability *p* < 0.05. All statistical analyses were performed by a senior professional statistician (A.B.) using SPSS-26 software (IBM, Armonk, NY, USA).

## 3. Results

Overall, we included 169 participants with aortic stenosis who underwent a TAVI procedure (86 males; 50.9%) with a median follow-up of 439 (318–585) days in this study. The resulting baseline data are presented on Table 1. As depicted on Table 1, baseline median EQ5D5L was 62 (IQR 45–83), KCCQ = 43 (IQR 33–52), and CFS = 4 (IQR 3–5).

### 3.1. Predictors for the Primary Endpoint (Midterm Mortality after TAVI)

**Figure 1:**
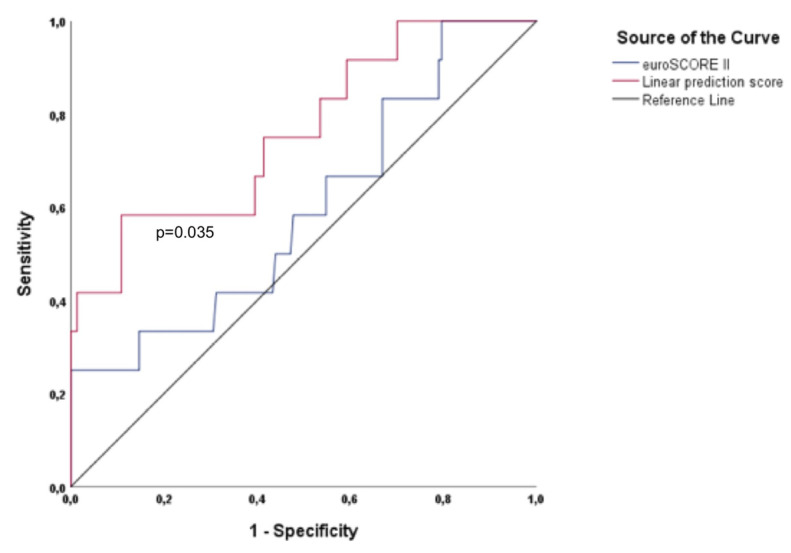
Shown is the area under the ROC curve for ESII and for the calculated linear prediction score 1 for the primary endpoint midterm mortality. The calculated linear prediction score (LPS) 1 (top) has a significantly higher (*p* = 0.035) area under the ROC curve compared with ESII (bottom).

As shown in Table 2, in addition to eGFR (based on cystatin C), NTproBNP, and ES II, KCCQ was also confirmed (being the only PROM) as a predictor of the primary endpoint by univariate analysis. Furthermore, KCCQ was found to be an independent predictor in the multivariate Cox regression hazard model, with an HR 0.976 (95% CI =0.953–0.999; *p* = 0.037) in addition to eGFR (based on cystatin C) (with an HR 0.977; 95% CI = 0.964–0.991; *p* = 0.001). As shown in Figure 1, the calculated LPS1 showed a significantly higher area under the ROC curve compared with ESII alone. Regarding the area under the curve (AUC) values for the primary endpoint, LPS1 demonstrated a significantly higher AUC value of 0.761 (*p* = 0.003; 95% CI = 0.612–0.911) compared to the AUC value of 0.597 of ESII (*p* = 0.264; 95% CI = 0.425–0.769). Moreover, quite significant AUC values were found for KCCQ (AUC = 0.739; *p* = 0.006; 95% CI = 0.597–0.881) and eGFR based on cystatin C (AUC = 0.732; *p* = 0.007; 95% CI = 0.587–0.878).

### 3.2. Predictors for the Secondary Endpoint LoS

**Figure 2:**
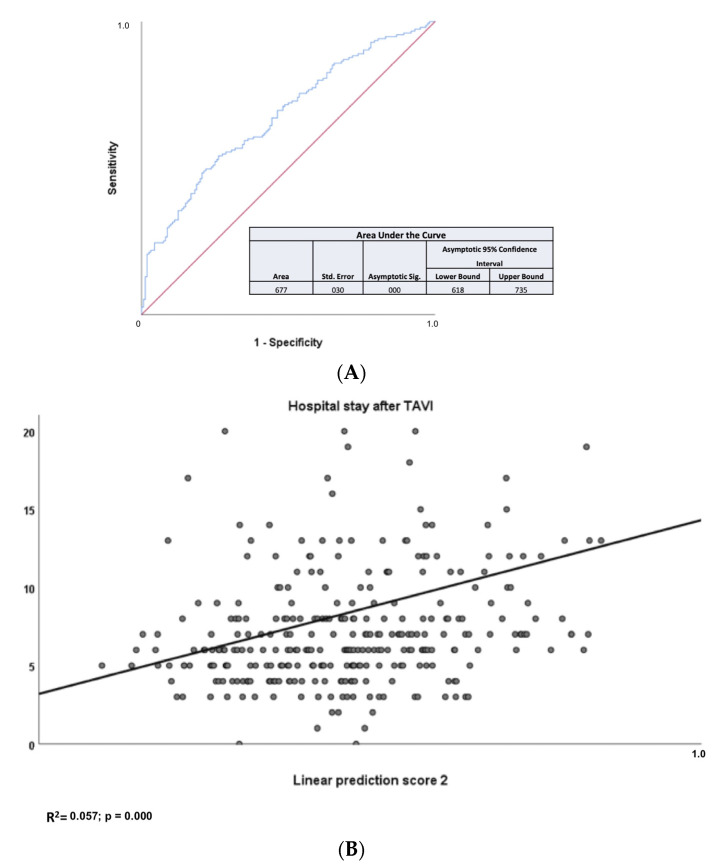
(**A**) Area under the ROC curve for the calculated LPS 2 for the secondary endpoint LoS. The calculated linear prediction score (LPS) 2 provided an area under the ROC curve of 0.667 (*p* < 0.001; 95% confidence interval 0.618–0.735). (**B**) Values for the secondary endpoint LoS as a function of LPS 2. Calculation of linear prediction score (LPS) 2 based on baseline levels of eGFR based on cystatin C and NTproBNP and EQ5D5L score allowed evaluation of LoS (R^2^ = 0.057; *p* < 0.001).

The median LoS was 6 days. Based on this finding, we have decided to choose this numerical cut-off value to better refine the secondary endpoint. As depicted in Table 2, two biomarkers, namely eGFR (based on cystatin C) and NTproBNP and two PROMs, namely KCCQ and EQ5D5L, were associated with longer hospital stay in the univariate analysis. More specifically, eGFR based on cystatin C (HR= 0.985; 95% CI =0.971–0.998; *p* = 0.028) and NTproBNP (HR= 1.557; 95% CI =1.035–2.343; *p* = 0.034) and EQ5D5L (HR= 0.985; 95% CI =0.975–0.994; *p* = 0.002) were found to be independent predictors for post-interventional stay in the multivariate analysis. As shown in Figure 2A, LPS 2 resulted in an area under the ROC curve of 0.667. Calculation of LPS2, based on baseline levels of eGFR (based on cystatin C) and NTproBNP and baseline EQ5D5L score, allowed evaluation of LoS (Figure 2B). According to these data, the HR for cystatin is < 1; this means that high values of eGFR (based on cystatin C) are associated with a protective effect and lower values are associated with poor prognosis. Low eGFR (based on cystatin C) levels, high NTproBNP levels, and low EQ5D5L values predicted a LoS > 6 days, which was the median LoS.

### 3.3. Predictors for the Secondary Endpoint Complications

A total of 80 patients (47.34%) developed 92 complications after the TAVI procedure (12 patients developed two complications each). Namely, 46 patients (27.22%) needed a cardiac pacemaker, 7 patients (4.14%) suffered a stroke, 5 patients (2.96%) developed an infection, 6 patients (3.55%) needed a cardiopulmonary resuscitation, 23 patients (13.61%) developed a vascular complication, 2 patients (1.18%) developed lower limb ischemia, and 3 patients (1.78%) developed other complications. None of the analyzed parameters could reliably predict any complication in the multivariate analysis. Only male sex was associated with the emergence of complications in the univariate analysis (see Table 2); ES II showed only a trend to be associated with the emergence of complications as shown in Table 2.

## 4. Discussion

We provide evidence that baseline KCCQ and eGFR (based on cystatin C) are independent predictors for midterm mortality after TAVI. Additionally, EQ5D5L score values, serum cystatin levels, and serum NTproBNP levels predict in-hospital stay. Therefore, among all tested PROMs, KCCQ proves beneficial to the prediction of mortality and EQ5D5L improves the prediction of in-hospital stay after trans-femoral TAVI. Male sex predicted complications only in the univariate analysis. These preliminary results lay the ground for personalized pre-interventional risk stratification and patient consultation based on the patients’ perspective and condition. They can also be used to adapt clinical management pathways according to personalized post-interventional risk and, therefore, may significantly promote patient safety.

This novel method, if confirmed in further studies, has the potential to be quite useful in everyday clinical practice in the future and may significantly strengthen the predictive value of the other more time-consuming objective measures required to generate the ESII. Thus, this method can play a significant additive role in supporting informed decisions on expected TAVI outcomes both from the patients’ and from the clinicians’ perspective. Therefore, from a clinical quality and safety perspective, important clinical decisions may be substantially supported by the predictive value added by certain PROMs (namely EQ5D5L and KCCQ, either separately or within the framework of a TAVI Scorecard). We suggest that these promising findings should be further validated in larger (multi-center) studies.

To the best of our knowledge, this is the first study that was prospectively designed to test KCCQ in combination with other patient-reported outcomes and/or clinical scoring systems or biomarkers as predictors of TAVI outcomes. In a similar study in patients with chronic heart failure, KCCQ was more sensitive to clinically meaningful changes in health status over time compared with NYHA class. The authors concluded that changes in KCCQ may have more prognostic value than changes in NYHA class [26].

Regarding the predictive value of eGFR (based on cystatin C), it should be noted that aortic stenosis (AS) and renal dysfunction share risk factors and often occur simultaneously. Although no other data—apart from these in our present study—after TAVI have been previously reported in the literature, severely impaired renal function (eGFR < 30 mL/min/1.73 m^2^) was independently associated with all-cause mortality in patients after surgical aortic valve replacement (AVR) [27]. Therefore, the findings of the present study provide strong evidence that the well-documented risk posed by chronic kidney disease on AVR risk may also exist in TAVI patients as well and should hence be considered in pre-interventional TAVI risk assessment.

Of note, based on our findings, blood levels of NTproBNP, known to be secreted by the myocardium in response to increased mechanical wall stress, appear to have mainly a short-term impact on TAVI outcomes and influence the acute post-TAVI physiological state. This is illustrated by their predictive effect on LoS while showing no impact on midterm mortality or complications. This finding should be further considered in future studies, because blood NTproBNP levels are known to show strong positive correlations with severity and symptom onset of aortic stenosis [28,29,30]. Accordingly, it would have been expected that NTproBNP levels would correlate with more chronic outcomes, such as all-cause midterm mortality (or even complications). However, no such correlations were found in our study. In contrast to other studies [12], we found no predictive value of baseline NTproBNP levels for midterm mortality but a predictive value for LoS. In addition, although hemoglobin levels at baseline have been previously reported to be predictive for 1-year mortality after TAVI [31], we could not confirm such a predictive value in our patient cohort.

It should be noted that our patients were much older than patients presented in other studies [32]. This may be a plausible reason for the emergence of more complications in our cohort compared with other studies that had included younger participants.

### 4.1. Strengths of the Study

This was a prospective study. Given that the study was conducted in a single medical center, there was no variability in the standard of provided care, which is an issue that could otherwise have confounded the results. The same group of clinicians/interventional cardiologists, with prior extensive relevant experience, performed only trans-femoral TAVI interventions. Patients were referred from a confined western European geographical region with diverse social and/or cultural backgrounds. Additionally, this is the first prospective study reporting on the additive predictive value of KCCQ and eGFR (based on cystatin C) on midterm mortality and on the additive predictive value of EQ5D5L on LoS after TAVI.

### 4.2. Study Limitations

This is a pilot single-center study and should therefore be considered hypothesis generating. Accordingly, the present findings should be interpreted with caution. Our study involved TAVI implants from various device providers/manufacturers ((Abbott, Chicago, IL, USA; Boston Scientific, Marlborough, MA, USA; Edwards, Irvine, CA, USA; Medtronic, Minneapolis, MN, USA) and hence, the specific implant type could have confounded the results. For this reason, we have performed an additional statistical analysis comparing the complication rate based on implant type; we have not found any association of the TAVI device or implant type with any intra- or post-procedural complications (*p* = 0.130).

### 4.3. Implications for Future Studies

To increase the external validity of the present findings, multi-center studies are warranted. In addition, the relevant predictors (KCCQ, EQ5D5L, eGFR based on cystatin C, blood NTproBNP levels, ESII) should be assessed at 30 days, one year, and two years after TAVI implantation in order to evaluate their additive predictive value for longer term outcomes, namely more than 2 years after TAVI. In addition, it may be useful to develop and prospectively evaluate risk-stratified clinical management pathways after TAVI based on these baseline predictors in order to increase patient safety both in the short-term and in the midterm. Similar to PROM findings reported in patients after myocardial infarction [33], the patients’ experience after TAVI (especially the one depicted by KCCQ and EQ5D5L) may yield novel endpoints for use in clinical trials.

## 5. Conclusions

This novel predictive method provides strong evidence that KCCQ significantly adds to the predictive value of eGFR (based on cystatin C) on midterm mortality while EQ5D5L significantly adds to the predictive value of NTproBNP on in-hospital stay after trans-femoral TAVI. Therefore, baseline PROMs significantly enhance personalized TAVI risk stratification, improve personalized pre-interventional patient education and counseling, and may help reduce post-TAVI risk.

## Figures and Tables

**Table 1:** Baseline characteristics of the study participants (number *N* = 169).

Baseline Characteristic	*N*	Range of Values	% of Total Participants
Male sex, *N* of participants	86		50.89% of all participants
Age, years	82.01	(78.23–84.54)	–
ES II, score values	4	(2.24–6.31)	–
NYHA I, *N* of participants	7		4.14% of all participants
NYHA II, *N* of participants	43		25.44% of all participants
NYHA III, *N* of participants	111		65.68% of all participants
NYHA IV, *N* of participants	8		4.73% of all participants
HB, g/dL	12.8	(11.6–13.7)	–
Creatinine, mg/dL	1.04	(0.86–1.29)	–
e-GFR based on cystatin C,	49.2	(35.1–62.5)	–
logNTProBNP	1.701	(0.564–3.896)	–
PCI, *N* of participants	90		53.25% of all participants
CAD, *N* of participants	125		73.96% of all participants
PM, *N* of participants	44		26.04% of all participants
AF, *N* of participants	97		57.4% of all participants
COPD, *N* of participants	53		31.36% of all participants
PAD, *N* of participants	74		43.79% of all participants
DM, *N* of participants	67		39.64% of all participants
Previous heart surgery, *N*	28		16.57% of all participants
CPR, *N* of participants	5		2.96% of all participants
Neurological dysfunction, *N*	27		15.98% of all participants
LoS after TAVI, days	6	(5–8)	–
CFS, score value	4	(3–5)	–
EQ5D5L, score value	62	(45–83)	–
KCCQ, score value	43	(33–52)	–

Baseline characteristics including comorbidities, clinical events, PROMs (patient-reported outcome measures), and LoS. The clinical events presented on this table are either included or not included in the calculation of the ESII Score. Values are median (interquartile range). ESII = EuroSCORE II; NYHA = New York Heart Association; HB = hemoglobin; e-GFR = estimated glomerular filtration rate (based on cystatin C); PCI = percutaneous coronary intervention, CAD = coronary artery disease; PM = pace-maker; AF = atrial fibrillation; COPD = chronic obstructive pulmonary disease; PAD = peripheral arterial disease; CPR = cardiopulmonary resuscitation; LoS = length of stay; DM = diabetes mellitus; CFS = clinical frailty scale; EQ5D5L = EuroQol − 5 Dimension − 5 Levels; KCCQ = Kansas City Cardiomyopathy Questionnaire.

**Table 2:** Univariate analysis of the predictors for primary and secondary endpoints.

Variable	HR (95% CI) Primary Endpoint Midterm Mortality	*p*-Value	HR (95% CI) Secondary Endpoint LoS > 6 days	*p*-Value	HR (95% CI) Secondary Endpoint Complications	*p*-Value
Male sex	1.29 (0.41–4.07)	0.662	0.77 (0.42–1.43)	0.412	**2.29 (1.09–4.85)**	**0.030**
Age	1.11 (0.99–1.25)	0.070	1.01 (0.97–1.05)	0.792	1.02 (0.97–1.07)	0.485
NYHA	1.11 (0.43–2.87)	0.830	1.06 (0.63–1.79)	0.820	0.85 (0.47–1.54)	0.585
HB	1.00 (0.74–1.36)	0.988	0.88 (0.74–1.04)	0.133	1.22 (0.98–1.51)	0.074
Creatinine	1.47 (0.99–2.18)	0.054	1.34 (0.88–2.03)	0.176	1.11 (0.74–1.67)	0.617
e-GFR	**0.96 (0.94–0.99)**	**0.001**	**0.98 (0.96–0.99)**	**0.000**	0.99 (0.98–1.02)	0.944
NTProBNP	**2.34 (1.42–3.86)**	**0.001**	**2.12 (1.48–3.02)**	**0.000**	0.76 (0.41–1.41)	0.387
CFS	1.14 (0.83–1.56)	0.405	1.25 (1.09–1.44)	0.002	0.91 (0.73–1.15)	0.444
EQ5D5L	0.98 (0.96–1.01)	0.134	**0.98 (0.97–0.99)**	**0.000**	1.01 (0.99–1.02)	0.346
KCCQ	**0.93 (0.89–0.98)**	**0.005**	**0.98 (0.96–0.99)**	**0.007**	1.01 (0.98–1.03)	0.691
ES II	**1.12 (1.06–1.18)**	**0.000**	1.04 (1.01–1.08)	0.016	1.03 (0.99–1.06)	0.064
AF	2.25 (1.11–4.56)	0.024	1.08 (0.67–1.74)	0.757	0.64 (0.29–1.42)	0.271
Infarct_bin	1.46 (0.65–3.27)	0.356	-	-	-	-
Cardio shock	2.51 (1.21–5.16)	0.013	-	-	-	-
CPR	0.05 (0.00–121)	0.557	-	-	-	-
COPD	2.10 (1.02–4.33)	0.044	-	-	-	-
PM	0.98 (0.34–2.80)	0.968	-	-	-	-
CAD	1.22 (0.59–2.51)	0.588	-	-	-	-
PCI	1.19 (0.59–2.43)	0.621	-	-	-	
DM	0.77 (0.34–1.72)	0.522	-	-	-	
PAD	1.95 (0.96–3.94)	0.064	-	-	-	
Neurol. Dysf.	1.80 (0.74–4.39)	0.196	-	-	-	
Ang. pectoris	0.64 (0.38–1.05)	0.079	-	-	-	

Presented are the results of the univariate analysis of each predictor for the primary and secondary endpoints. Hazard ratio with 95% confidence intervals and respective *p*-values are depicted. HR = hazard ratio; Infarct bin = myocardial infarct binary (yes, no), Cardio shock = history of fully compensated cardiogenic shock (>3 months before TAVI), other abbreviations as in Table 1.

## Data Availability

Data supporting the reported results may be provided by the authors upon reasonable request.
